# Current landscape of the immunoproteasome: implications for disease and therapy

**DOI:** 10.1038/s41420-025-02698-0

**Published:** 2025-08-25

**Authors:** Zifeng Zou, Yanglin Hao, Zetong Tao, Weicong Ye, Zilong Luo, Xiaohan Li, Ran Li, Kexiao Zheng, Jiahong Xia, Chao Guo, Xi Zhang, Jie Wu

**Affiliations:** Department of Cardiovascular Surgery, Union Hospital, Tongji Medical College, Huazhong University of Science and Technology, Wuhan, China

**Keywords:** Cardiovascular diseases, Immunopathogenesis

## Abstract

The immunoproteasome, an inflammation-induced proteasome variant, coordinates proteostasis and adaptive immunity by replacing constitutive subunits (β1, β2, β5) with inducible counterparts (β1i, β2i, β5i). This specialization enhances antigen processing for MHC class I presentation and oxidative protein clearance. Beyond immune regulation, it critically contributes to cardiovascular, respiratory, neurodegenerative, autoimmune, retinal, and oncological pathologies through mechanisms involving NF-κB activation, mitochondrial dysfunction, and inflammatory polarization. While β5i-specific inhibitors (e.g., ONX 0914) show therapeutic potential in preclinical models by mitigating proteotoxicity and inflammation, the immunoproteasome’s dual roles—cytoprotective or pathogenic—are context-dependent, necessitating precise targeting strategies. This review synthesizes recent advances in immunoproteasome biology, disease mechanisms, and therapeutic prospects, while highlighting unresolved questions on subunit specificity and microenvironmental regulation.

## Facts

- Immunoproteasomes swap standard subunits for interferon-induced ones, enhancing antigen presentation and oxidative stress defense during inflammation.
- Immunoproteasomes play dual roles in diseases, which provide cytoprotective functions during myocardial ischemia while driving inflammatory cascades in atherosclerosis and neurodegenerative disorders.
- Immunoproteasome-selective inhibitors curb cytokine storms and proteotoxic stress while sparing constitutive proteasome activity, offering promise for autoimmune and cardiovascular diseases.

## Open questions

- What molecular mechanisms link immunoproteasome activity to metabolic stress pathways and organ-specific autophagy networks?
- What biomarkers can reliably measure the therapeutic efficacy of immunoproteasome inhibition across diseases?

## Introduction

Cellular protein homeostasis is fundamentally regulated by the ubiquitin-proteasome system (UPS), a highly conserved machinery responsible for the selective degradation of damaged or misfolded proteins. Ubiquitination, a post-translational modification mediated by a cascade of enzymes (E1, E2, and E3), directs substrates to either proteasomal degradation via K48-linked polyubiquitin chains or to autophagy-lysosomal pathways through K63-linked chains. This process governs critical cellular processes, including cell cycle progression [1], apoptosis [2, 3], stress responses [4] and signal transduction [5]. Within this framework, the immunoproteasome emerges as a dynamic and inflammation-adapted variant of the constitutive proteasome [6, 7].

The immunoproteasome is distinguished by the replacement of standard catalytic subunits (β1, β2, β5) with inducible counterparts (β1i/LMP2, β2i/MECL-1, β5i/LMP7), which are upregulated by pro-inflammatory cytokines, such as interferon-gamma (IFN-γ) and tumor necrosis factor-alpha (TNF-α) [8]. This structural adaptation enhances proteolytic efficiency, particularly in generating antigenic peptides for presentation on major histocompatibility complex (MHC) class I molecules, thereby bridging innate and adaptive immunity [9]. Beyond its canonical role in antigen processing, the immunoproteasome acts as a critical stress sensor, clearing oxidatively damaged proteins and maintaining proteostasis under pathological conditions such as viral infections and oxidative stress [10].

However, the immunoproteasome exhibits a dualistic nature: while it safeguards cellular integrity under acute stress, its dysregulation can exacerbate inflammatory cascades and tissue damage. Emerging evidence underscores its pivotal involvement in diverse pathologies, including cardiovascular diseases [11], respiratory disease [12], neurodegenerative disorders [13], retinal disease [14], cancer progression [15], and autoimmune conditions [16].

Pharmacological targeting of immunoproteasome subunits, such as β5i-specific inhibitors (e.g., ONX 0914), has shown therapeutic promise in preclinical models by restoring proteostasis and dampening maladaptive immune responses [17]. Nonetheless, its context-dependent roles—protective versus detrimental—vary across tissues and disease stages, highlighting the need for precise therapeutic strategies. This review synthesizes recent advances in immunoproteasome biology, elucidates its multifaceted contributions to disease pathogenesis, and evaluates the translational potential of subunit-specific interventions, while addressing unresolved questions regarding microenvironmental regulation and functional heterogeneity.

## Composition and type of proteasome

### Proteasome

**Fig. 1:**
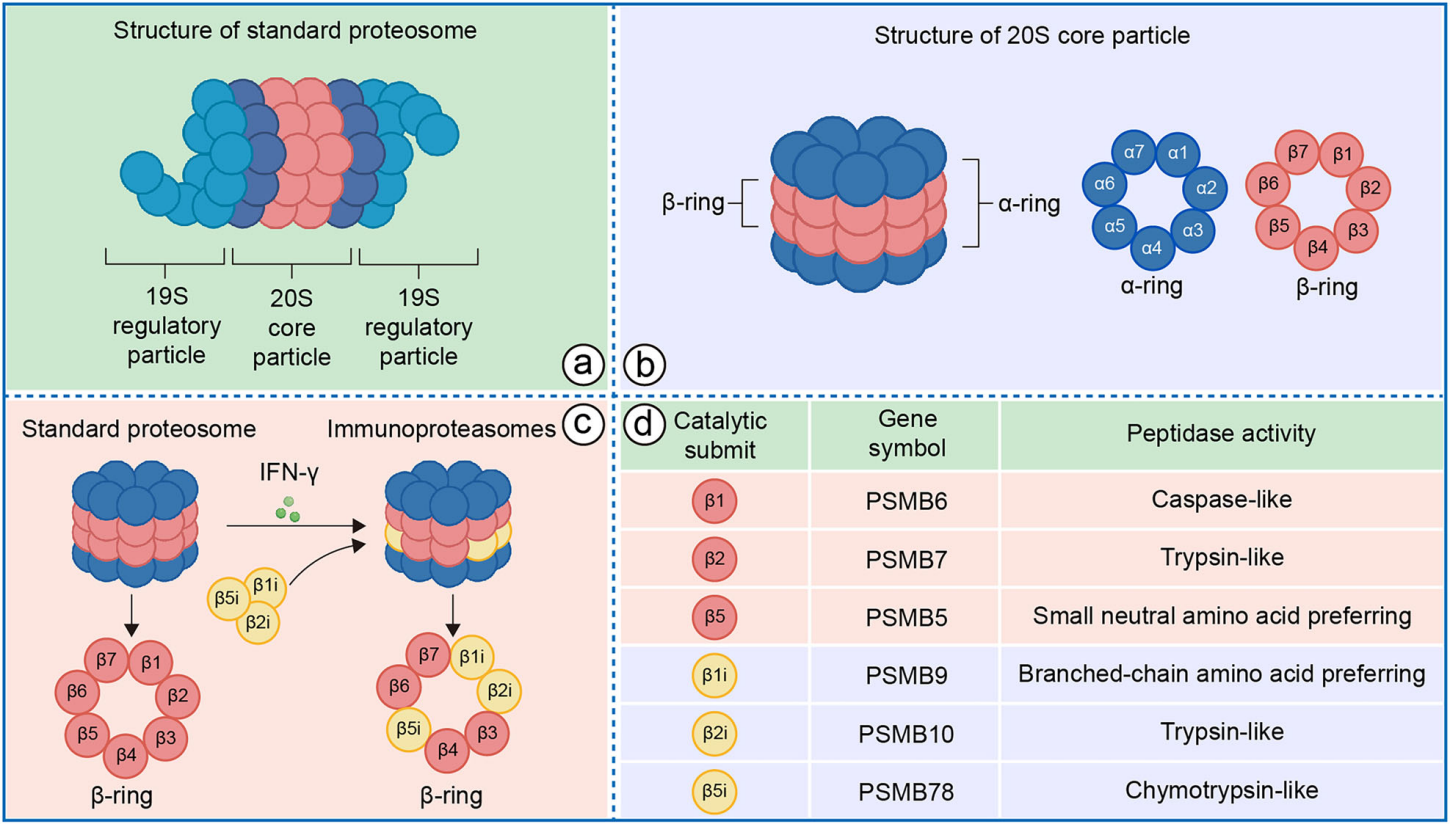
Structure and composition of the proteasome and immunoproteasome. **a** Architecture of the 20S core particle (CP) and 19S regulatory particle (RP). **b** The 20S CP comprises α-rings (substrate entry) and β-rings (catalytic sites). **c** Subunit substitution defines immunoproteasome (IP) specificity: IFN-γ induces replacement of constitutive β1/β2/β5 subunits with inducible β1i (LMP2), β2i (MECL-1), and β5i (LMP7). **d** The table summarizes the gene symbols and peptidase activities of the catalytic subunits in both the SP and IP.

Cellular protein homeostasis is maintained through selective proteolysis mediated by the proteasome, an ATP-dependent molecular machine that degrades ubiquitin-tagged proteins [18]. In eukaryotic cells, the 26S proteasome, a 2.5 MDa complex comprising at least 32 distinct subunits, consists of two functionally distinct subcomplexes: the 20S core particle (CP) and the 19S regulatory particle (RP), which caps one or both ends of the CP (Fig. 1a) [19–21]. Protein degradation occurs within the narrow proteolytic chamber of the CP, which has a barrel-like cylindrical structure composed of four stacked heptameric rings of α and β subunits, with a stoichiometry of α_1-7_β_1-7_β_1-7_α_1-7_ (Fig. 1b) [22]. The outer α-rings form a gated entryway through tightly interwoven N-terminal tails, restricting access to the internal proteolytic chamber within the β-rings, thus ensuring precise degradation control. Among the seven β subunits, only β1, β2, and β5 possess catalytic proteolytic activity, exhibiting caspase-like, trypsin-like, and chymotrypsin-like activities (Fig. 1d), respectively. These activities are critical for the specific degradation of substrates [23–25].

The 19S RP, comprising 19 subunits, is organized into lid and base subcomplexes. The lid contains nine non-ATPase subunits, including the deubiquitinating enzyme Rpn11, which is critical for efficient substrate processing by removing ubiquitin tags [26]. The base includes a heterohexameric ring of six AAA+ ATPases, Rpt1–Rpt6, which drive ATP-dependent substrate unfolding and translocation through a narrow pore into the CP [27]. The ATPases’ carboxy termini interact with specific α-ring sites to open the gate [28]. Additionally, the base incorporates non-ATPase subunits Rpn1, Rpn2, and ubiquitin receptors Rpn10 and Rpn13, with Rpn1 mediating recruitment of ubiquitin shuttle receptors (Rad23, Ddi1, Dsk2) and the non-essential deubiquitinase Ubp6 [29, 30]. The coordinated interplay between the RP’s substrate recognition, deubiquitination, and translocation functions and the CP’s gated proteolysis ensures the proteasome’s precision and efficiency in regulating protein turnover, a process essential for cellular function and homeostasis [31–33].

### Immunoproteasome

The immunoproteasome is a specialized type of proteasome, constitutively expressed in hematopoietic cells and widely distributed across immune tissues, where it plays a pivotal role in immune responses [34]. It can also be induced in non-immune cells by pro-inflammatory cytokines (such as IFN-γ, IFN-α, IFN-β) and tumor necrosis factor (TNF), with IFN-γ being the most potent inducer [35, 36]. In response to inflammatory factor, the immunoproteasome is induced through the activation of STAT1 and IRF1, leading to the replacement of the proteolytic subunits β1, β2, and β5 in the proteasome core particle (CP) with immune-specific subunits β1i (LMP2), β2i (MECL-1), and β5i (LMP7) (Fig. 1c) [37]. This specialized immunoproteasome exhibits higher chymotrypsin-like and trypsin-like activities, enabling it to more efficiently degrade various antigenic proteins [38, 39]. The assembly of the immunoproteasome is notably rapid—approximately four times faster than that of the constitutive proteasome—due to the direct binding of β5i to the assembly chaperone POMP (also known as UMP1), facilitating a swift response to immune and inflammatory stimuli [38].

Beyond the 19S regulatory subunit, additional types of regulatory subunits have been identified. Notably, the immunoproteasome’s principal regulatory subunit is PA28, comprising three isoforms: PA28α, PA28β, and PA28γ [40]. The IFN-γ-inducible PA28αβ heterodimer binds to the 20S CP, inducing conformational changes in the α subunits that open the gated channels, thereby enhancing substrate entry and peptide release [41]. This structural adaptation retains substrates in the 20S antechamber prior to degradation, significantly augmenting the efficiency of antigen presentation [42, 43]. The immunoproteasome’s catalytic and regulatory components can assemble into three distinct configurations: the 26S proteasome (19S-20S-19S), which supports ubiquitin-dependent and ATP-dependent proteolysis; the PA28-capped proteasome (PA28-20S-PA28), which facilitates ATP-independent antigen processing; and the hybrid proteasome (PA28-20S-19S), which integrates both regulatory mechanisms to optimize protein degradation for immune surveillance [44]. This structural diversity underscores the immunoproteasome’s specialized role in generating antigenic peptides, enhancing the precision and adaptability of the adaptive immune response.

## Function and expression of immunoproteasome in immunocytes

Homeostasis is a critical determinant of cellular lifespan. The maintenance of cellular protein homeostasis is achieved through a balance between protein stability and stress resistance, protein repair, protein refolding, and the proteolysis of damaged proteins [45]. The ubiquitin-proteasome system (UPS) is pivotal in this process, orchestrating the selective degradation of proteins tagged with ubiquitin, a highly conserved polypeptide [46–48]. Since its initial description decades ago, UPS has been widely recognized for playing a crucial role in regulating nearly all biological processes within the cell. It is not only essential for maintaining the homeostasis of proteins and amino acids but also involved in regulating multiple fundamental cellular processes, including the cell cycle, DNA replication, transcription, signal transduction, and stress responses [18, 20, 34]. These processes involve an enzymatic cascade of E1, E2, and E3 enzymes that conjugate ubiquitin to target proteins, forming polyubiquitin chains with distinct topologies that dictate substrate fate [49, 50]. Chains linked via lysine 48 (K48) primarily signal proteasomal degradation, whereas K63-linked chains direct damaged organelles, such as ribosomes, endoplasmic reticulum, or mitochondria, to the autophagy-lysosome pathway [51, 52].

Under normal physiological conditions, the standard proteasome maintains cellular homeostasis by efficiently degrading proteins. However, in inflammatory states—such as those triggered by interferon-gamma (IFN-γ)-induced oxidative stress or infections with viruses, fungi, or bacteria—this machinery is superseded by the immunoproteasome, a specialized variant adapted to heightened cellular demands [8]. Research in mice demonstrates that, following infection, standard proteasomes in affected organs are swiftly replaced by newly synthesized immunoproteasomes, underscoring their critical role in such contexts [53–56]. The immunoproteasome excels at processing antigenic proteins, generating peptides for presentation on major histocompatibility complex (MHC) class I molecules—a pivotal step in activating adaptive immunity [57]. Therefore, under non-inflammatory conditions, the standard proteasome efficiently handles protein degradation, whereas the specialized function of the immunoproteasome is more pronounced in inflammatory environments [45]. In cells lacking key immunoproteasome subunits (such as LMP7), inflammatory conditions tend to lead to the accumulation of polyubiquitinated and oxidized proteins, exacerbating cellular stress and tissue damage. This phenomenon indicates that the immunoproteasome plays an irreplaceable role in responding to inflammation-induced protein homeostasis stress [58].

Beyond protein degradation, studies in mice with immunoproteasome subunit knockouts reveal its broader influence on immune regulation. It shapes the composition and activation of lymphocyte subpopulations, drives the maturation and differentiation of B cells and Th17 cells, and modulates macrophage polarization [59]. These effects correlate with altered transcriptional profiles and cytokine expression in immune cells, emphasizing the immunoproteasome’s integral role in maintaining homeostasis across both adaptive and innate immune systems [60, 61]. These findings demonstrate that the immunoproteasome plays an important role in maintaining homeostasis of both adaptive and innate immune cells. The specific functions and expressions of immunoproteasome and subunits in various immune cells are listed in Table 1.

**Table 1:** Expression and function of the immunoproteasome in immune cells.

Immune cells	Immunoproteasome subunits	Expression	Function	Pathways involved	Ref
Dendritic cells (DCs)	β1i (LMP2), β2i (MECL-1), β5i (LMP7)	High expression in professional antigen-presenting cells (APCs)	Antigen processing and presentation via MHC class I; Regulation of gene expression, signaling, and maturation	MHC class I pathway; STAT1/IRF1 signaling	[34, 64, 104]
Macrophages	β1i, β2i, β5i	Constitutive and inducible expression, especially in alveolar macrophages	Antigen processing and presentation; Inflammatory signaling (NF-κB activation) Polarization (M1 vs. M2)	NF-κB pathway; STAT1/STAT3 balance for polarization	[34, 39, 61, 100]
Neutrophils	β1i, β2i, β5i	Potential role in antigen presentation	Possible involvement in MHC class II expression under certain conditions	MHC class II pathway	[101]
NK cells	β1i, β2i, β5i	Indirect regulation via MHC class I expression on target cells	Protection of cells from NK cell-mediated killing during infection	MHC class I pathway	[102]
T cells	β1i, β2i, β5i	Constitutive expression	Clonal expansion and survival during immune activation; Regulation of T cell repertoire and differentiation	ERK signaling; Proteostasis maintenance	[34, 59, 103, 107, 108]
B cells	β1i, β2i, β5i	Constitutive expression	Maturation and differentiation; Antibody class switching (via MHC-II presentation)	ERK signaling; MHC class II pathway	[60, 103]

### Innate immunocytes

#### Antigen processing and presentation

**Fig. 2:**
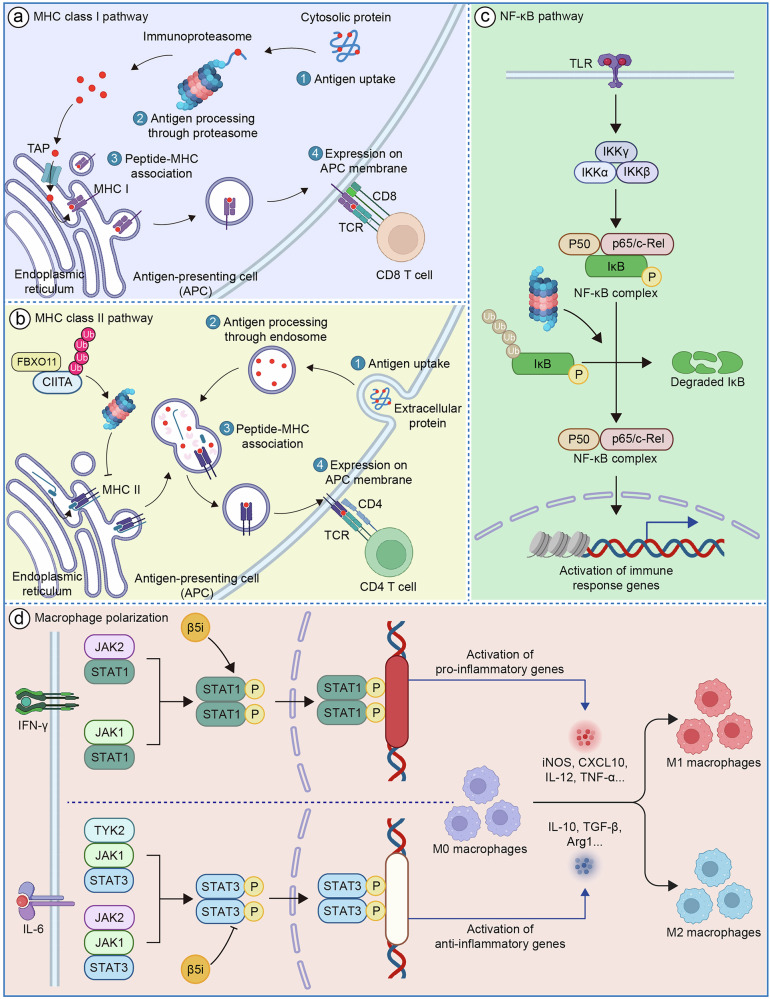
Immunoproteasome-mediated regulation of cellular immune responses. **a** MHC class I antigen presentation: IP processes cytosolic antigens into peptides transported via TAP to the ER for MHC I loading, activating CD8⁺ T cells. β5i/LMP7 ensures hydrophobic anchor residues critical for MHC I binding. **b** The immunoproteasome degrades CIITA via FBXO11-mediated targeting, suppressing MHC class II expression. **c** NF-κB activation: IP degrades IκB, releasing NF-κB (p65/p50) to induce pro-inflammatory cytokines. β5i promotes sustained NF-κB signaling in cardiovascular and autoimmune pathologies. **d** Macrophage polarization: IFN-γ-induced β5i enhances STAT1 activation, favoring pro-inflammatory M1 over anti-inflammatory M2 phenotypes, a mechanism implicated in atherosclerosis and fibrosis.

Innate immune cells, including dendritic cells, macrophages, and natural killer cells, form the body’s frontline defense against pathogens, while antigen-presenting cells (APCs) orchestrate cell-mediated adaptive immunity through molecular recognition via major histocompatibility complex (MHC) class I molecules. One of the key mechanisms is that antigen-presenting cells present antigenic peptides via MHC class I molecules, a process mediated by the proteolytic complex of the immunoproteasome [62]. Structurally and sequentially similar to the standard proteasome, the immunoproteasome collaborates in producing peptides for MHC class I presentation, with its distinct hydrolytic activity enhancing the efficiency and diversity of antigen presentation, thereby facilitating robust immune responses [63]. Due to its unique structure and hydrolytic activity, the immunoproteasome significantly enhances the presentation function of MHC class I antigenic peptides during the induction process. The antigen processing pathway for MHC class I-restricted epitopes from cytosolic proteins begins with ubiquitin-mediated degradation by the immunoproteasome’s 20S core, producing peptide fragments that are subsequently refined by aminopeptidases and translocated to the endoplasmic reticulum (ER) via TAP-1 and TAP-2 transporters. In the ER, ERAP1 or ERAP2 may further trim peptide N-termini to optimize binding to MHC class I molecules. Chaperones, including calnexin, calreticulin, and tapasin, facilitate proper folding of the MHC class I α-chain and β2-microglobulin, enhancing the peptide-binding groove’s receptivity. Tapasin then promotes the association of the MHC class I complex with TAP-1/2, enabling suitable peptides to occupy the groove, leading to the dissociation of the heavy chain/peptide/β2m complex from chaperones and its transport to the plasma membrane (Fig. 2a). Additionally, peptides from membrane-associated or secreted proteins generated in the ER further diversify the MHC class I peptide repertoire [64]. Various proteasome subtypes efficiently initiate antigen processing, generating peptides of 8 or 9 residues tailored to fit the peptide-binding groove of nascent MHC class I molecules in the endoplasmic reticulum [65]. The MHC class I groove preferentially accommodates peptides with hydrophobic C-terminal anchor residues, primarily produced by the chymotrypsin-like activity of the immunoproteasome’s β5/LMP7 subunit. A shift in LMP2’s proteolytic activity toward chymotrypsin-like function further enhances the production of such peptides, increasing the diversity of sequences available for MHC class I binding [66–68]. Although other cytosolic proteases contribute to peptide generation, the immunoproteasome remains the primary source of peptides with hydrophobic C-termini, optimizing MHC class I-mediated immune responses.

Research demonstrates that in β5i-deficient mice, MHC class I expression on cell membranes is markedly reduced, indicating impaired intracellular antigen presentation. Similarly, in immunoproteasome knockout mice, MHC class I expression is approximately halved compared to wild-type controls, underscoring the immunoproteasome’s critical role in efficient antigen presentation [69]. In contrast, unlike MHC class I, MHC class II molecules are expressed exclusively on specialized antigen-presenting cells, presenting exogenous antigens processed in lysosomes, such as those from phagocytosed pathogens [70, 71]. The NLR protein CIITA (Class II Transactivator) acts as the master regulator of MHC class II gene transcription [72]. FBXO11, a member of the F-box protein family with a length of 927 amino acids [73], specifically targets CIITA for ubiquitin-mediated degradation by the immunoproteasome in immune cells, thereby modulating CIITA levels and decreasing MHC class II expression (Fig. 2b) [74].

Dendritic cells and macrophages can cross-present extracellular antigens on MHC class I molecules through both proteasome-dependent and proteasome-independent pathways [75–78]. The primary peptide source for MHC class I presentation is defective ribosomal products (DRIPs), resulting from errors during protein synthesis [79–81]. DRIPs are rapidly ubiquitinated at the ribosome and degraded in situ by the proteasome. Infections or inflammation, often triggered by IFN signaling, enhance protein synthesis, thereby increasing DRIP formation and peptide availability [82]. Therefore, IP function relies on the clearance of inflammation-induced DRIPs and damaged proteins, improving the peptide supply for antigen presentation [58]. This capability optimizes dendritic cell cross-presentation of tumor and viral exogenous antigens, thereby promoting CD8⁺ T cell activation and bolstering immune responses against infections and malignancies. For dendritic cells (DC), the immunoproteasome not only facilitates antigen processing but also broadly regulates gene expression, signaling pathways, and maturation. Its absence impairs DC antigen presentation, leading to immune dysfunction and a macrophage-like phenotype, underscoring its non-redundant role in maintaining DC-specific functions within the immune system [83].

#### Regulation of inflammatory signaling pathways

The immunoproteasome critically regulates the NF-κB signaling pathway, a key modulator of inflammatory cytokine gene transcription [84]. The NF-κB family, comprising NF-κB1, NF-κB2, and type II proteins RelA, RelB, and c-Rel, typically remains inactive in cells [85]. NF-κB activation relies on immunoproteasome-mediated degradation of I-κB, an inhibitor that sequesters NF-κB in the cytoplasm (Fig. 2c) [86]. Phosphorylation of I-κB triggers its ubiquitination and subsequent degradation, releasing NF-κB to translocate to the nucleus and induce pro-survival gene expression [87, 88]. In innate immune cells, this immunoproteasome-driven mechanism accelerates production of inflammatory cytokines, such as TNF-α and IL-6, enhancing early pathogen responses.

#### Cell activation and function maintenance

Macrophages are the most plastic cells in the hematopoietic system, present in all tissues, and exhibit a wide functional diversity. They play roles in development, homeostasis, tissue repair, and immunity [89]. Tissue-resident macrophages are present in most tissues, with general functions and tissue-specific functions that can be described as auxiliary. Resident macrophages integrate signals from a wide range of environmental sensors to coordinate adaptive cellular responses, which is crucial for the growth, remodeling, and homeostasis of specialized tissue cells [90]. For example, alveolar macrophages are highly specialized tissue-resident macrophages that reside on the respiratory surface of the lungs. These cells express particularly high levels of immunoproteasomes in the lungs, which are crucial for defending the lung host against inhaled pathogens and resolving tissue damage [91–94]. During acute infections or injury, alveolar macrophages are activated to a classical M1 phenotype and may secrete pro-inflammatory cytokines to initiate the recruitment of inflammatory cells [95]. After the acute inflammatory outbreak, alveolar macrophages can clear cell debris and apoptotic inflammatory cells through a process known as efferocytosis and, in response to IL-4/-13 or IL-10 signals, polarize to an alternatively activated M2 macrophage phenotype [96]. Alveolar macrophages enter an anti-inflammatory state characterized by metabolic changes, activation of anti-inflammatory signaling factors, expression of M2 marker genes, and the production of anti-inflammatory and pro-growth cytokines [97–99]. The absence of the immunoproteasome subunit LMP7 leads to enhanced STAT3 signaling and weakened STAT1 signaling, thereby promoting the polarization of macrophages toward the M2 (anti-inflammatory) phenotype. Conversely, intact immunoproteasome function supports STAT1 activity, favoring the M1 (pro-inflammatory) phenotype, indicating that the immunoproteasome influences macrophage polarization by regulating the STAT1/STAT3 balance (Fig. 2d) [100].

For neutrophils, related studies are limited, but as innate immune cells, they may influence antigen presentation and inflammatory responses through the immunoproteasome. Studies show that neutrophils can express MHC class II molecules under certain conditions, potentially involving the function of the immunoproteasome [101]. For NK cells, the immunoproteasome primarily regulates their function indirectly by affecting MHC class I expression on target cells. Studies indicate that cells with immunoproteasome deficiencies are more likely to become targets of NK cells during infection, suggesting a role in protecting cells from NK cell-mediated killing [102].

### Adaptive immunocyte

In contrast to professional and non-professional antigen-presenting cells that necessitate immunoproteasome expression for effective antigen processing, T cells and B cells exhibit constitutive immunoproteasome expression [103].

As mentioned above, the immunoproteasome generates peptide fragments with hydrophobic C-termini by degrading intracellular proteins, which are then loaded onto MHC class I molecules and presented on the cell surface for recognition and activation by CD8 T cells. This is a critical immune surveillance mechanism against virally infected cells and tumor cells. Meanwhile, mice with a knockout of the immune subunit β5i exhibit significant changes in MHC class I antigen presentation, affecting the response of CD8 T cells to viral infections [104]. The immunoproteasome subunit LMP7 has been shown to modulate adaptive immune cells: LMP7 deficiency or catalytic inhibition compromises the differentiation capacity of naive CD4^+^ T helper (Th) cells toward Th1/Th17 lineages, while simultaneously promoting regulatory T cell differentiation through modified cellular signaling pathways [59]. Foxp3 is the core transcription factor of Tregs, and its expression and stability are influenced by multiple regulatory factors [105]. Eos, a member of the Ikaros family, is known to interact with Foxp3 and, under certain conditions (e.g., inflammatory environments), suppress Foxp3 activity or affect downstream gene expression in Tregs; while Eos is redundant in the suppressive function of Tregs, it limits pro-inflammatory responses in conventional T cells by inhibiting IL-2 and CD40L expression [106]. The immunoproteasome may maintain Treg stability and suppressive function by degrading Eos (a potential inhibitor of Foxp3).

Moreover, T cells derived from mice with immunoproteasome subunit deficiencies demonstrate compromised proliferative capacity and reduced survival upon transfer into virally infected wild-type recipients. These findings cannot be explained by antigen presentation-mediated graft rejection, instead revealing that the immunoproteasome intrinsically regulates T cell clonal expansion and survival during immune activation [107, 108]. Mice lacking LMP2 exhibit diminished B cell populations along with reduced circulating CD4^+^ and CD8^+^ T cell counts [60]. MECL-1 knockout mice demonstrate altered T cell repertoires [109], while combined deficiency of MECL-1 and LMP7 results in T cell hyperproliferation [110]. Additionally, chemical inhibition (as opposed to genetic deletion) of LMP7 compromises ERK signaling sustainability and triggers moderate proteotoxic stress, exerting distinct effects on T and B lymphocyte functionality and viability, by significantly reducing the antigen processing and MHC-II presentation capacity of B cells, leading to impaired CD4⁺ T cell activation, decreased secretion of cytokines (e.g., IL-4 and IL-21), and thereby suppressing antibody class switching (e.g., IgG1 and IgE) [103].

## Immunoproteasomes and diseases

**Fig. 3:**
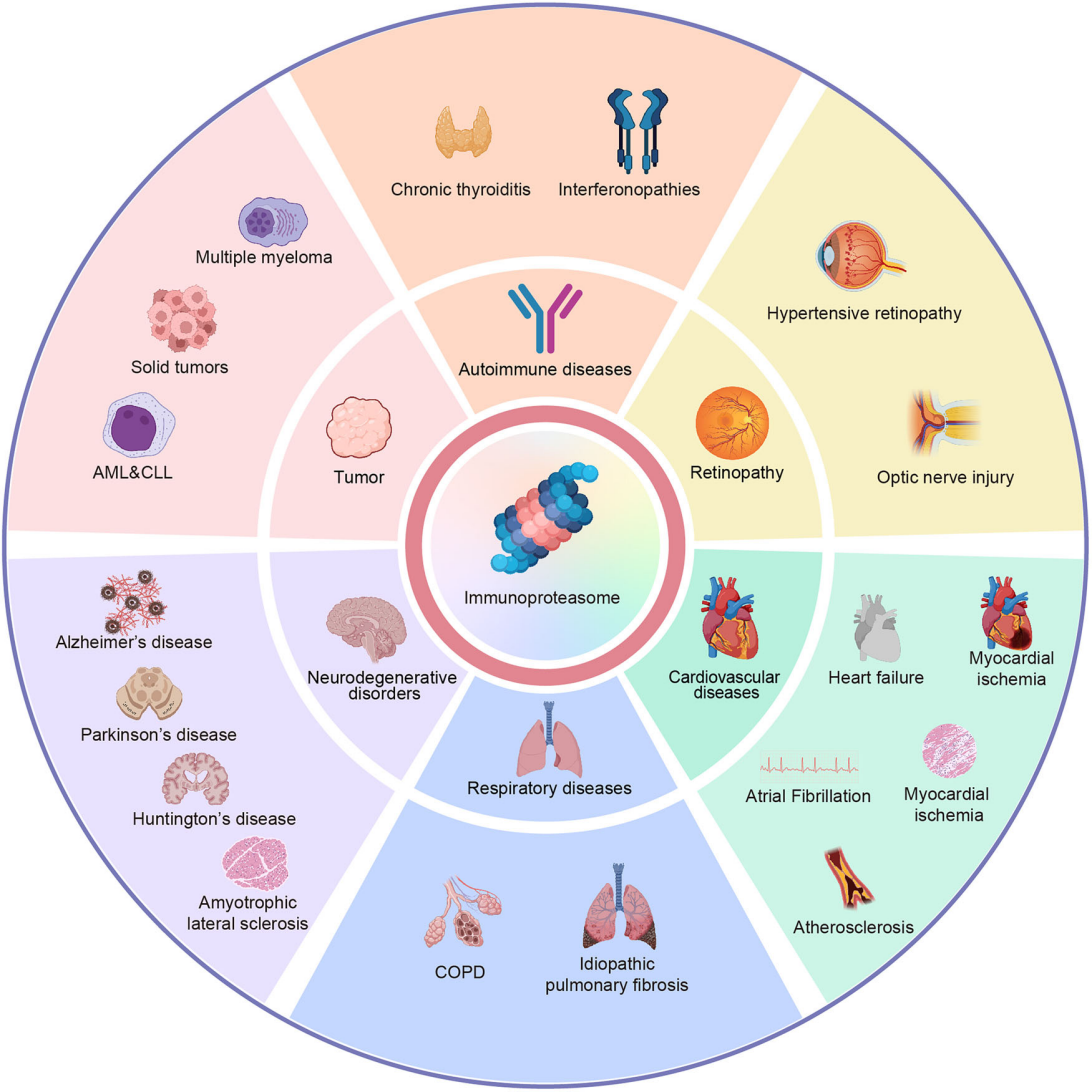
Diseases associated with immunoproteasome dysregulation. The immunoproteasome contributes to pathogenesis across multiple systems through subunit-specific mechanisms: cardiovascular diseases, respiratory diseases, neurodegenerative disorders, autoimmune diseases, retinal diseases and tumors.

The immunoproteasome plays a pivotal role in a wide array of diseases, exhibiting context-dependent functions that range from protective to pathogenic. Its involvement spans cardiovascular diseases, respiratory diseases, neurodegenerative disorders, retinal diseases, tumor, and autoimmune disorders (Fig. 3). The specific role of immunoproteasome and subunits in various diseases is listed in Table 2.

**Table 2:** Immunoproteasome in diseases.

Category	Disease	Immunoproteasome subunits	Mechanistic role	Pathways involved	Therapeutic implications	Ref
Cardiovascular diseases	Heart failure	β5i (LMP7)	Exacerbates hypertrophy via ATG5-mediated autophagy; Degrades ATRAP, promoting RAAS activation	AKT, ERK, MAPK, STAT3; PTEN regulation	β5i inhibition (e.g., PR-957) attenuates hypertrophy	[11, 114, 115]
	Atrial Fibrillation	β5i, β2i (PSMB10)	Promotes fibrosis and inflammation via NF-κB and TGF-β/Smad pathways; Degrades PTEN and ATRAP	NF-κB pathway, TGF-β/Smad; AKT1 signaling	β5i inhibition (e.g., ONX 0914) or β2i knockout reduces AF	[117, 118, 120]
	Myocardial ischemia	β1i, β2i, β5i	Protects against I/R injury by regulating mitochondrial dynamics and oxidative stress	PP2A-AMPK-PGC1α; Keap1-NRF2; PTEN-Akt	β5i activation (e.g., MK-886) mitigates oxidative stress	[121, 122, 124, 125]
	Myocarditis	β1i, β2i, β5i	Clears damaged proteins, but also exacerbates inflammation	NF-κB pathway, cytokine production	β5i inhibition (e.g., ONX 0914) reduces inflammation	[128–131]
	Atherosclerosis	β1i, β2i, β5i	Promotes inflammation and foam cell formation; Suppresses efferocytosis	NF-κB pathway, ICAM1/VCAM1 expression	β5i or β2i inhibition reduces plaque formation	[86, 133, 135, 136]
Respiratory diseases	COPD	β1i, β2i, β5i	Involved in protein homeostasis and inflammation; Activates adaptive immune responses	Unfolded protein response; MHC class I pathway	Potential target for reducing inflammation	[144–146]
	Idiopathic pulmonary fibrosis	β1i, β2i, β5i	Upregulates MHC-I via cGAS/STING pathway, activating CD8^+^ T cells	cGAS/STING/IFN-I pathway	Inhibition may reduce T cell activation	[16]
Neurodegenerative disorders	Alzheimer’s disease (AD)	β5i (LMP7), β1i (LMP2)	Increases around Aβ plaques, promoting neuroinflammation; LMP7 regulates cytokine secretion in microglia	NF-κB pathway	LMP2/LMP7 inhibitors (e.g., YU102) improve cognitive function	[148–150, 156]
	Parkinson’s disease (PD)	β5i	Degrades α-synuclein aggregates; LMP7 upregulated in PD brains	NRF2-mediated POMP regulation	Enhancing immunoproteasome activity may reduce α-syn aggregation	[152, 153]
	Huntington’s disease	β1i, β2i, β5i	Upregulated in neurons, associated with neurodegeneration	Unknown	Potential target to modulate proteostasis	[154]
	Amyotrophic lateral sclerosis	β1i, β2i, β5i	Induced in astrocytes and microglia, possibly via TNF-α/IFN-γ	Cytokine-mediated pathways	Role in neuroinflammation; potential therapeutic target	[155]
Retinal diseases	Retinopathy	β5i, β2i(LMP10)	Promotes vascular permeability and angiogenesis via degradation of ATRAP/PTEN/ATG5	NF-κB pathway, TGF-β/Smad, AKT/mTORC1, VEGF	β5i/LMP7 inhibition reduces pathological angiogenesis	[14, 157–160]
Tumor	Various cancers	β1i, β2i, β5i	Processes tumor antigens for MHC-I presentation; Regulates NF-κB and p53 pathways	NF-κB pathway; p53 degradation	Immunoproteasome inhibitors may enhance anti-tumor immunity or induce cancer cell death	[161, 162, 172, 180]
	Acute myeloid leukemia (AML)	β5i(PSMB8)	Stabilizes oncogenic networks via K63 ubiquitination; Degrades tumor suppressors via K48 ubiquitination	BASP1-KMT2A axis, UBE2N-mediated proteostasis	PSMB8 inhibitors synergize with Menin inhibitors	[167–169]
	Chronic lymphocytic leukemia (CLL)	β5i(LMP7)	Confers resistance to BCL-2 inhibitors via p38 MAPK activation	p38 MAPK signaling	Combined β5i inhibitors + venetoclax	[170, 171]
Autoimmune diseases	Systemic autoinflammatory diseases (SAID)	β1i, β2i, β5i	Mutations lead to persistent innate immune activation	Type I interferon signaling	Immunoproteasome modulation may reduce autoinflammation	[186–188]
	Chronic thyroiditis	β1i, β2i, β5i	IFN-γ induces immunoproteasome, contributing to thyroid inflammation	IFN-γ signaling	Potential target for reducing thyroid inflammation	[189, 190]

### Cardiovascular diseases

#### Heart failure

Heart failure is a complex clinical syndrome characterized by impaired cardiac pumping ability, which is insufficient to meet the metabolic demands of the body’s tissues and organs. The etiology of heart failure is multifactorial, with common causes including hypertension, coronary artery disease, diabetes, obesity, and cardiomyopathy [111]. Heart failure is associated with numerous signaling pathways and molecules, including the regulation of metabolism and protein synthesis by PI3K/AKT/mTOR and AMPK; calcium signaling pathways, such as CaMKII and PLC-IP3, which promote pathological hypertrophy; NO/cGMP/PKG signaling, which inhibits pathological processes; RAAS and catecholamines inducing remodeling through Gq and β-adrenergic receptors; ROS and NF-κB exacerbating oxidative stress and inflammation; and PTEN on chromosome 10, among others [112]. The immunoproteasome plays an important regulatory role, especially in the development of heart failure induced by myocardial hypertrophy. Studies have shown that the immunoproteasome regulates the ATG5-mediated autophagy process via β5i and exacerbates myocardial hypertrophy and heart failure through signaling pathways such as AKT, ERK, and inflammatory factors [11]. Autophagy inhibits myocardial hypertrophy by hydrolyzing IGF1R and gp130, leading to a reduction in the phosphorylation levels of AKT, mTOR, JAK2, STAT3, and ERK1/2 [113]. Additionally, the knockout of β5i or treatment with the β5i-specific inhibitor PR-957 has been shown to ameliorate cardiac hypertrophy in the DOCA-salt hypertensive mouse model through activation of PTEN [114].

On the other hand, the renin-angiotensin-aldosterone system (RAAS) is another key factor influencing the development of myocardial hypertrophy. Ang II promotes hypertrophy by enhancing myocardial remodeling, whereas AT1R-associated protein (ATRAP) binds to the AT1 receptor (AT1R) to prevent the overactivation of RAAS. The immunoproteasome degrades ATRAP, thereby promoting AT1R activation, and activates the MAPK and STAT3 pathways, ultimately leading to myocardial hypertrophy [115].

In myocardial remodeling research, the immunoproteasome subunit low molecular weight protein (LMP-2) has been shown to play a critical role in myocardial adaptive remodeling. In adult rat ventricular cardiomyocytes (ARVC), LMP-2 expression is significantly upregulated during the early stages of adaptation to two-dimensional culture conditions or pressure overload (such as hypertension) and integrates into the proteasome to enhance protein degradation ability. This process relies on the activation of the p38 MAPK signaling pathway; inhibition of LMP-2 expression or this pathway significantly weakens cardiomyocyte survival and adaptive capacity [116].

#### Atrial fibrillation

Recent studies have broadened the understanding of the immunoproteasome’s pathological role in atrial fibrillation (AF). In an Angiotensin II (Ang II)-induced AF model, the activity of the immunoproteasome is significantly increased, as evidenced by the upregulation of catalytic subunits β5i (PSMB8) and β2i (PSMB10). β5i enhances AT1 receptor activity by promoting ATRAP (Ang II type 1 receptor-associated protein) degradation, activating NF-κB and TGF-β/Smad signaling pathways, which in turn aggravates atrial fibrosis and inflammation [117, 118].

Additionally, the natural compound gallic acid (GA) inhibits immunoproteasome activity, prevents PTEN degradation, and suppresses downstream AKT1 signaling, significantly improving Ang II-induced AF and atrial remodeling [119]. Similarly, melatonin acts as an inhibitor of the immunoproteasome, stabilizing ATRAP levels and alleviating Ang II-induced AF along with associated pathological changes such as fibrosis, inflammation, and oxidative stress [118]. These studies collectively indicate that the immunoproteasome plays a central regulatory role in atrial electrical remodeling, structural remodeling, and inflammation.

In another study, the knockout of the immunoproteasome subunit β2i (PSMB10) effectively reduces the occurrence and duration of Ang II-induced AF, and mitigates atrial fibrosis and inflammation. Mechanistically, the absence of PSMB10 reduces PTEN degradation, inhibits AKT1 signaling activation, and blocks the TGF-β/Smad pathway and NF-κB-mediated inflammatory cascade [120].

#### Myocardial ischemia

The immunoproteasome and its subunits play a crucial role in cardiac protection against myocardial ischemia/reperfusion (I/R) injury through the regulation of various molecular mechanisms.

Ursolic acid improves mitochondrial biogenesis and dynamic balance by upregulating the expression and activity of immunoproteasome subunits and activating the PP2A-AMPK-PGC1α signaling pathway [121]. This regulation further alleviates oxidative stress and myocardial cell apoptosis induced by I/R injury. Similarly, the β2i subunit regulates the Parkin-Mfn1/2-mediated mitochondrial fusion process, inhibiting excessive mitochondrial fission and ultimately protecting myocardial function [122]. Notably, the small molecule TCH-165 enhances immunoproteasome activity, promotes the degradation of mitochondrial fission protein Drp1, and upregulates the expression of fusion proteins Mfn1/2, further optimizing mitochondrial dynamics [123]. These studies collectively emphasize the central role of the immunoproteasome in regulating mitochondrial function, with specific mechanisms closely linked to the dynamics of mitochondrial fission and fusion.

In addition to mitochondrial regulation, the immunoproteasome protects the myocardium from I/R injury by modulating key signaling pathways of oxidative stress. MK-886 enhances the activity of the β5i subunit, promotes the degradation of Keap1, and activates the NRF2-dependent antioxidant response, thereby effectively mitigating I/R-associated oxidative stress and apoptosis [124]. Moreover, recent research has revealed the critical role of the LMP2 (β1i) subunit in ischemic preconditioning (IPC) by mediating PTEN protein degradation and activating the downstream Akt signaling pathway, thereby enhancing myocardial protection [125]. These findings indicate that the function of the immunoproteasome in signal transduction and cell protection is closely linked to the specificity of its subunits.

Additionally, sRAGE upregulates the activity of the β1i and β5i subunits by inducing IFN-γ expression, thereby inhibiting p53 protein accumulation and I/R-induced apoptosis[126]. Correspondingly, Chen and colleagues demonstrated that immunoproteasome subunit expression influences not only cardioprotection but also the inflammatory and repair mechanisms in other tissues (e.g., brain tissue) [127]. Collectively, these findings highlight the cooperative role of the immunoproteasome in protecting multiple organs.

In summary, the immunoproteasome and its subunits play multifaceted, multi-level roles in the regulation of myocardial I/R injury by modulating mitochondrial dynamics, oxidative stress, and key signaling pathways (such as PP2A-AMPK, Keap1-NRF2, and PTEN-Akt).

#### Myocarditis

In the pathogenesis of myositis, the immunoproteasome exerts diverse functions by both augmenting antigen presentation and viral clearance, and by helping to sustain protein homeostasis. However, the immunoproteasome is also involved in exacerbating the inflammatory response, leading to secondary cellular damage [69]. Multiple mouse models of myocarditis have demonstrated elevated levels of β1i, β5i, and β2i expression, along with increased proteolytic activity and an upregulated antigen processing system [128]. O Research by Opitz et al. indicates that mice lacking the β5i (LMP7) subunit exhibit more severe myocardial damage and inflammation following Coxsackievirus B3 (CVB3) infection. This aggravated pathology is associated with disrupted protein homeostasis and oxidative stress, and the immunoproteasome protects the myocardium from inflammatory cytotoxicity by removing polyubiquitinated damaged proteins [129]. Similarly, the immunoproteasome-specific inhibitor ONX 0914 can effectively alleviate CVB3-induced myocarditis by reducing myocardial monocyte/macrophage infiltration and suppressing excessive production of proinflammatory cytokines and chemokines, thereby decreasing inflammation and improving cardiac function [130].

In autoimmune myocarditis models, the immunoproteasome has also been demonstrated to be a crucial regulator of pathological processes. Bockstahler et al. demonstrated that either knocking out the three catalytic subunits of the immunoproteasome (LMP2, LMP7, MECL1) or administering ONX 0914 can significantly reduce CD4^+^ T cell-mediated inflammatory responses and fibrosis in experimental autoimmune myocarditis. Moreover, such intervention shifts the equilibrium between effector and regulatory T cells, upregulating inhibitory molecules like PD-1, which in turn mitigates myocardial injury and enhances cardiac performance [131].

Additional research reveals that the modulation of systemic inflammatory responses by the immunoproteasome might impact the course of myocarditis. ONX 0914 mitigates acute viral myocarditis by inhibiting a systemic inflammatory storm, indicating that systemic immune regulation is the main mechanism by which this drug exerts its protective action [132]. In summary, the immunoproteasome plays multiple roles in both the pathogenesis and treatment of myocarditis by regulating inflammatory responses, maintaining protein homeostasis, and preventing cytotoxic damage to cardiomyocytes, thereby providing a theoretical basis for the development of immunoproteasome-targeted therapeutic strategies.

#### Atherosclerosis

Similarly, the immunoproteasome plays an important role in cardiovascular inflammatory responses. Evidence suggests that the immunoproteasome is intimately associated with the advancement of inflammation in atherosclerotic lesions, the formation of foam cells, and plaque instability.

The long non-coding RNA PSMB8-AS1 induces the expression of PSMB9 (LMP2), markedly increasing ICAM1 and VCAM1 levels in endothelial cells, which strengthens monocyte adhesion to the endothelium and intensifies vascular inflammation and atherosclerosis [133]. In addition, LMP7 (β5i) suppresses MERTK-dependent clearance of apoptotic cells, resulting in enlarged necrotic cores and intensified inflammation in atherosclerotic lesions, thereby indicating a pro-inflammatory role for LMP7 in lesion progression [86].

It has been demonstrated that the genetic deletion or pharmacological inhibition of the immunoproteasome can markedly alleviate the severity of atherosclerotic lesions. For instance, while β5i generally plays a pro-inflammatory role in inflammatory diseases, its deletion has a relatively minor effect on protein homeostasis in some models, suggesting its role may vary across different inflammatory contexts [134]. In contrast, the deletion of LMP10 (β2i) in the ApoE-deficient model markedly reduced M1 macrophage infiltration in arterial lesions, while enhancing the proportion of M2 anti-inflammatory macrophages, thereby suppressing atherosclerotic lesions [135]. Research indicates that the immunoproteasome inhibitor ONX-0914 effectively suppresses early plaque formation and inflammatory responses by reducing the activation of pro-inflammatory macrophages and T cells, while improving indicators related to metabolic syndrome, such as blood lipids and glucose levels [136].

Additionally, it has been shown that PSMB9 upregulates the transcription factor ZEB1, which in turn promotes the expression of ICAM1 and VCAM1, thereby intensifying vascular inflammation and atherosclerotic lesions [137]. This finding provides new insights for targeting PSMB9 in treatment, suggesting that inhibiting PSMB9 may become a potential strategy for treating cardiovascular diseases.

#### Others

The immunoproteasome is associated with various cardiovascular diseases, with its subunits acting as risk or protective factors in several contexts. During myocardial ischemia-reperfusion (I/R) injury, the immunoproteasome exerts protective effects by modulating oxidative stress and apoptosis pathways. Studies have found that inhibition of the immunoproteasome exacerbates myocardial cell damage after reperfusion, while the LMP7 subunit reduces I/R-associated oxidative stress and apoptosis by activating the Keap1-NRF2 antioxidant pathway [127].

Immunoproteasome dysfunction is also a critical factor in the pathogenesis of diabetic cardiomyopathy (DCM). Overexpression of the LMP7 subunit in diabetic hearts exacerbates endothelial-to-mesenchymal transition (EndMT) in myocardial cells, leading to increased myocardial fibrosis. Pharmacological inhibition of LMP7 activity significantly alleviates myocardial fibrosis and restores heart function [138]. Likewise, bioinformatics analysis has further established PSMB8 (β5i) as a key immune molecular marker of DCM, closely associated with metabolic disturbances and inflammatory conditions in diabetic hearts [139]. Moreover, in a doxorubicin (Dox) induced cardiotoxicity model, downregulation of immunoproteasome subunits increases myocardial cell apoptosis, while upregulation of β1i, β2i, and β5i expression significantly reduces cardiotoxicity and protects heart function [140].

In inflammatory vascular diseases like atherosclerosis, the immunoproteasome modulates macrophage pyroptosis and inflammatory pathways. LMP7 induces macrophage pyroptosis by activating the NF-κB pathway, thereby promoting the formation of atherosclerosis [141]. In abdominal aortic aneurysm (AAA), inhibition of the immunoproteasome significantly reduces the infiltration of inflammatory cells and associated tissue damage [141, 142].

However, current research on the immunoproteasome has certain limitations. For example, although its function has been validated in various cardiovascular diseases, the specific mechanisms of its action in different pathological states are not fully understood, particularly its dual role in dynamic inflammatory environments [139, 143]. Future research needs to further clarify the functional differences of the immunoproteasome in specific diseases and different tissue microenvironments in order to better develop targeted therapeutic strategies against the immunoproteasome.

### Respiratory diseases

Recent studies have pointed out that mechanisms involving changes in proteostasis, such as the unfolded protein response, endoplasmic reticulum stress, and inhibition of the ubiquitin-proteasome system, are key in the pathogenesis of COPD [144, 145]. This dysfunctional protein processing ultimately leads to the accumulation of nonfunctional and potentially cytotoxic proteins, with misfolded proteins contributing to alveolar cell apoptosis, inflammation, and the typical autophagy seen in COPD. The circulating immunoproteasome response to these antigens is activated, inducing changes in the adaptive immune population, such as increased T cell differentiation through MHC-I-TCR interactions, expansion of Th17 cells, (autoreactive) B cells, and downregulation of regulatory T cells, leading to inflammation, lung tissue damage, and autoimmune phenomena in COPD patients [146].

Additionally, mitochondrial DNA stress responses upregulate the immunoproteasome and MHC I antigen presentation pathway via the cGAS/STING/type I interferon pathway, leading to spontaneous activation of CD8^+^ T cells. In patients with idiopathic pulmonary fibrosis, chronic activation of adaptive immune responses induced by abnormal lung epithelial cells via the cGAS/STING pathway is closely associated with the activation of CD8^+^ T cells in the affected lung tissue [16].

### Neurodegenerative disorders

Neurodegenerative diseases such as Alzheimer’s disease (AD), Parkinson’s disease (PD), Huntington’s disease (HD), and amyotrophic lateral sclerosis (ALS) are characterized by the accumulation of abnormal proteins and the activation of neuroinflammation. The immunoproteasome, as a specialized proteasome isoform, plays a key role in regulating inflammatory responses and proteostasis.

The core pathology of AD includes the deposition of amyloid β (Aβ), tau protein aggregation, and the activation of neuroinflammation [147]. Studies have shown that the immunoproteasome plays an important role in the progression of AD pathology. Reactive astrocytes and microglial cells exhibit increased immunoproteasome activity around the Aβ plaque regions. Upregulation of the immunoproteasome is closely associated with the release of inflammatory factors, suggesting its regulatory role in AD-related neuroinflammation [148]. Further studies have shown that LMP7 deficiency significantly alters the cytokine secretion pattern in microglial cells and improves cognitive function in AD mice [149]. Moreover, LMP2 inhibitors such as DB-310 show significant cognitive improvement in AD models, highlighting the potential of LMP2 as a therapeutic target for AD [150].

The pathological feature of PD is the abnormal aggregation of α-synuclein (α-syn), forming Lewy bodies [151]. Overexpression of α-syn inhibits the expression of POMP regulated by the nuclear factor NRF2, leading to defects in immunoproteasome assembly, exacerbating α-syn aggregation and neurodegeneration [152]. Further studies confirm that the immunoproteasome helps maintain proteostasis by degrading α-syn aggregates, and its LMP7 subunit is significantly upregulated in the brain tissue of PD patients [153].

In HD models, the expression of LMP2 and LMP7 is significantly increased in neurons, accompanied by upregulation of immunoproteasome activity, which is closely associated with neurodegenerative changes [154]. Additionally, studies have found that in ALS mouse models, induction of the immunoproteasome primarily occurs in astrocytes and microglial cells, possibly mediated by cytokines such as TNF-α and IFN-γ [155].

The immunoproteasome may either play a protective role or exacerbate neuroinflammation in neurodegenerative diseases. For example, the immunoproteasome maintains cellular homeostasis by degrading damaged proteins, but its excessive activation may lead to the overrelease of inflammatory factors, further damaging neurons [148]. Dual inhibitors of LMP2 and LMP7, such as YU102, significantly reduce the release of inflammatory factors and improve cognitive function in AD mice, without affecting Aβ deposition [156].

### Retinal diseases

Recent studies have elucidated the pivotal role of immunoproteasomes in retinal pathologies, demonstrating their regulation of critical signaling pathways. Immunoproteasome subunits, such as LMP10 and β5i (LMP7), exhibit upregulated expression in various retinal disease models, modulating disease progression by degrading specific regulatory proteins. In hypertensive retinopathy, β5i (LMP7) promotes the degradation of ATRAP, activating downstream NF-κB, TGF-β/Smad, and ERK/Akt signaling pathways via AT1R, which exacerbates vascular permeability and inflammation [157]. Similarly, LMP10 (β2i) degrades PTEN, activating the AKT/IKKβ pathway and inducing IκBα phosphorylation and NF-κB target gene expression, thereby aggravating Ang II-induced retinal pathology [158]. Furthermore, in oxygen-induced retinopathy models, β5i suppresses autophagy by binding and degrading the key autophagy protein ATG5, promoting VEGF-mediated pathological angiogenesis [14]. These findings highlight the immunoproteasome’s role in disrupting protein homeostasis networks, such as the PTEN-ATRAP-ATG5 axis, during retinal disease progression.

In optic nerve injury models, depletion of immunoproteasome subunits LMP2 and LMP7 significantly delays retinal ganglion cell apoptosis, potentially by suppressing aberrant Akt signaling and reducing oxidative stress [159]. Additionally, mTORC1 signaling induces expression of the immunoproteasome subunit Psmb9 (β1i), accelerating the degradation of cell cycle proteins during retinal development, thereby regulating the proliferation and differentiation dynamics of neural progenitor cells [160]. This interplay between protein synthesis and degradation underscores the dual role of immunoproteasomes in maintaining retinal homeostasis.

Collectively, immunoproteasomes regulate NF-κB, Akt/mTORC1, and autophagy pathways by degrading key regulatory proteins such as ATRAP, PTEN, and ATG5, playing a central role in retinal inflammation, vascular remodeling, and neurodegeneration. Targeting specific immunoproteasome subunits or their downstream effectors may offer novel therapeutic strategies for retinal diseases.

### Tumor

In the process of tumorigenesis, several reports have documented dysregulated expression and function of the immunoproteasome [161]. Experiments have confirmed that the proteasome plays a critical role in cancer cell survival and cell cycle progression. Mutations in cancer cell genomes lead to the production of aberrant proteins and proteotoxic stress, making the clearance of accumulated proteins essential for cell survival; the increased protein synthesis must be matched by enhanced protein degradation to maintain proteostasis. Consequently, cancer cells often overexpress proteasome subunits, and inhibition of the proteasome can result in cancer cell death [162, 163]. The immunoproteasome is highly expressed in many cancers, including multiple myeloma, breast cancer, prostate cancer, and certain hematologic malignancies. Lymphocytes infiltrating the tumor microenvironment secrete large amounts of IFN-γ, which induces cancer cells to express the immunoproteasome [161, 164]. This phenomenon is particularly evident in solid tumors such as breast and colorectal cancers [165, 166].

High immunoproteasome expression can be achieved via non-IFN-γ pathways in certain hematologic cancers, such as acute myeloid leukemia (AML) [161]. In AML, immunoproteasomes regulate protein homeostasis and transcriptional networks, critically influencing leukemia cell survival and drug resistance. Studies reveal that AML cells with KMT2A rearrangements (KMT2A-r) exhibit specific dependence on the immunoproteasome catalytic subunit PSMB8 (β5i). Inhibiting PSMB8 upregulates the transcription factor BASP1, suppressing oncogenic gene expression driven by KMT2A fusion proteins, and synergizes with Menin inhibitors to enhance anti-leukemic activity [167]. Additionally, UBE2N-mediated K63 ubiquitination stabilizes oncogenic protein networks to maintain protein homeostasis in AML, whereas UBE2N inhibition triggers immunoproteasome-dependent K48 ubiquitination and degradation pathways, highlighting the bidirectional regulatory role of immunoproteasomes in AML [168]. Notably, AML cells can evade T-cell immunotherapy by downregulating immunoproteasome subunits (e.g., β1i), but targeting alternative epitopes (e.g., WT1₃₇₋₄₅) generated by constitutive proteasomes can overcome this resistance [169].

In contrast, immunoproteasome activity in CLL is markedly elevated compared to constitutive proteasomes and is closely associated with disease progression. CLL cells are highly sensitive to selective immunoproteasome inhibitors, such as LU035i, which induce apoptosis by targeting the β5i subunit with minimal toxicity to normal hematopoietic cells [170]. However, interferon-γ (IFN-γ) in the CLL microenvironment activates the p38 MAPK signaling pathway, promoting immunoproteasome formation and conferring resistance to the BCL-2 inhibitor venetoclax. Combined treatment with p38 MAPK inhibitors or immunoproteasome inhibitors (e.g., ONX-0914) reverses this resistance and enhances venetoclax cytotoxicity [171]. These findings elucidate the divergent roles of immunoproteasomes in AML and CLL, providing a foundation for developing tailored therapeutic strategies for distinct leukemia subtypes.

The NF-κB pathway is one of the key tumor signaling pathways regulated by the immunoproteasome [172]. In the classical pathway, activation of the IκB kinase complex (IKKβ/α/γ) leads to phosphorylation of the NF-κB inhibitor IκB, resulting in the ubiquitination of IκBα and its recognition and degradation by the proteasome [173–176]. When the immunoproteasome degrades IκB, NF-κB translocates into the nucleus, thereby promoting gene transcription and cell proliferation [177]. In addition to regulating this pathway, the immunoproteasome has been shown to process tumor antigens, influencing immune surveillance and immune evasion. However, its precise role in tumorigenesis and invasion remains unclear, and the underlying mechanisms are not fully understood [178, 179].

The p53 protein is an important transcription factor and serves as a key tumor suppressor [180]. In approximately half of human cancers, the p53 gene is mutated or deleted. The p53 protein is degraded by the 26S proteasome through a polyubiquitination process. Under normal conditions, the half-life of p53 is short, but under stress conditions (such as DNA damage), its degradation is inhibited, leading to a rapid increase in its levels. Inhibiting proteasome-mediated p53 degradation may become a promising tumor suppressive strategy [88, 180].

### Autoimmune diseases

The immunoproteasome participates in the development of various autoimmune diseases by regulating T cell polarization, the NF-κB signaling pathway, and the production of inflammatory cytokines by macrophages [181–185]. For example, inborn errors of immunity (IEI) primarily manifest as sterile autoinflammation and are often classified as systemic autoinflammatory diseases (SAID). SAID is usually caused by mutations in various pathways involving innate immune responses, which remain persistently activated after being triggered, leading to the development of systemic autoinflammation [186, 187]. SAID can be classified based on the primary inflammatory pathway it interferes with, one of which is called interferonopathies, characterized by enhanced type I interferon signaling, with proteasomal molecular defects closely linked to this pathological state [188].

The immunoproteasome is also involved in the pathogenesis of chronic thyroiditis. Transgenic mice with specific IFN-γ expression in the thyroid exhibit symptoms of chronic thyroiditis and hypothyroidism [189, 190]. These studies provide important theoretical support for the clinical application of the immunoproteasome as a potential therapeutic target and offer new insights into understanding the pathophysiological mechanisms of systemic inflammatory diseases.

## Immunoproteasoma-related therapeutic strategies

### Inhibitors

Bortezomib is a non-selective proteasome inhibitor that has been approved for the treatment of multiple myeloma [191]. In a phase I clinical trial involving patients with refractory hematologic malignancies, bortezomib demonstrated efficacy in patients with multiple myeloma, mantle cell lymphoma, and non-Hodgkin lymphoma. Phase II studies focused on relapsed and refractory myeloma, with about one-third of patients responding to treatment, leading to accelerated approval of bortezomib by the US FDA in 2003 as a treatment option [192]. Subsequently, bortezomib was further approved as a first-line treatment for newly diagnosed multiple myeloma [88]. However, due to the non-selective nature of bortezomib, it may affect other targets in addition to inhibiting the proteasome, leading to peripheral nervous system adverse effects in over 30% of patients [193].

As most proteasome inhibitors are non-selective, researchers have developed the selective β5i inhibitor PR-957/ONX0914, whose selectivity can be explained by the differences in the S1 pockets of β5 and β5i [194]. PR-957 shows at least 14 times higher selectivity for β5i compared to β5, while not significantly affecting the overall function of the proteasome. In animal models, PR-957 successfully blocked the presentation of β5i-specific antigens and inhibited the release of IL-23, IFN-γ, and IL-2, demonstrating its potential therapeutic effects [195]. Recent studies have found that the LMP7-selective inhibitor KZR-329 and the LMP2-selective inhibitor KZR-504 show significant effects in a rat aortic transplant model. By co-inhibiting LMP2 and LMP7, the generation of inflammatory cytokines, complement, and antibodies can be effectively suppressed, reducing apoptosis of vascular wall cells and significantly slowing the progression of transplant-associated arteriosclerosis [196].

### Nanomaterials

Extracellular vesicles (EVs) are nanoscale particles with a bilayer lipid membrane, containing various biomolecules such as proteins, lipids, mRNA, miRNA, DNA, and other small molecules [197, 198]. Mesenchymal stem cells (MSCs) are a promising cellular resource for treating a variety of diseases [199]. Currently, bortezomib (BTZ)-based chemotherapy regimens are the first-line treatment for multiple myeloma [200]. However, this regimen is associated with significant side effects, such as peripheral neuropathy, nephrotoxicity, and leukopenia. By encapsulating pH-responsive BTZ-containing polymeric nanoparticles in apoptotic MSC-derived extracellular vesicles (apoVs) and combining the synergistic effects of BTZ and apoVs, the therapeutic efficacy against multiple myeloma can be significantly enhanced [201].

## Conclusion and perspective

The immunoproteasome, an inflammation-induced proteasome variant, leverages its distinct catalytic subunits to play critical roles in antigen processing and proteostasis under stress conditions. Its functions in disease exhibit duality: it offers protection in conditions such as cardiovascular diseases, respiratory diseases, neurodegenerative disorders, autoimmune diseases, retinopathy, and cancer by mitigating cellular damage and clearing misfolded proteins; however, its dysregulation can exacerbate pathological progression via inflammatory signaling.

Despite progress, immunoproteasome research faces significant challenges. Subunit-specific functions, particularly in non-immune tissues, remain poorly understood; for instance, β5i/LMP7 drives NF-κB activation in cardiovascular pathologies, whereas β2i/MECL-1 influences macrophage polarization, yet the mechanisms are elusive. Additionally, microenvironmental regulation—via cytokine gradients, metabolic conditions, or autophagy interactions—is insufficiently characterized, impeding targeted therapy development. Preclinical studies, often reliant on genetic knockouts or non-specific inhibitors, fail to reflect subunit nuances in human diseases. Moreover, the long-term impacts of immunoproteasome modulation, including risks of immune suppression or off-target effects, remain underexplored clinically.

Future efforts should emphasize subunit-selective drug design to optimize therapeutic outcomes while preserving physiological proteostasis. Techniques such as cryo-EM and single-cell omics could clarify the structural and functional diversity of immunoproteasomes across tissues. Multi-omics approaches may elucidate how microenvironmental factors shape immunoproteasome activity in specific diseases. Clinically, rigorous trials are essential to evaluate the safety and efficacy of therapies like β5i inhibitors (e.g., ONX 0914) and nanomaterial-based delivery systems, particularly for chronic inflammatory and neurodegenerative conditions.
